# TCDD Toxicity Mediated by Epigenetic Mechanisms

**DOI:** 10.3390/ijms19124101

**Published:** 2018-12-18

**Authors:** Barbara Patrizi, Mario Siciliani de Cumis

**Affiliations:** 1 National Institute of Optics-National Research Council (INO-CNR), Via Madonna del Piano 10, 50019 Sesto Fiorentino, Italy; 2 European Laboratory for Non-Linear Spectroscopy (LENS), Via Nello Carrara 1, 50019 Sesto Fiorentino, Italy; 3 Italian Space Agency Contrada Terlecchia snc, 75100 Matera, Italy; mario.sicilianidecumis@asi.it

**Keywords:** dioxins, 2,3,7,8-TCDD, epigenetic mechanisms, environmental xenobiotics

## Abstract

Dioxins are highly toxic and persistent halogenated organic pollutants belonging to two families i.e., Polychlorinated Dibenzo-*p*-Dioxins (PCDDs) and Polychlorinated Dibenzo Furans (PCDFs). They can cause cancer, reproductive and developmental issues, damage to the immune system, and can deeply interfere with the endocrine system. Dioxins toxicity is mediated by the Aryl-hydrocarbon Receptor (AhR) which mediates the cellular metabolic adaptation to these planar aromatic xenobiotics through the classical transcriptional regulation pathway, including AhR binding of ligand in the cytosol, translocation of the receptor to the nucleus, dimerization with the AhR nuclear translocator, and the binding of this heterodimeric transcription factor to dioxin-responsive elements which regulate the expression of genes involved in xenobiotic metabolism. 2,3,7,8-TCDD is the most toxic among dioxins showing the highest affinity toward the AhR receptor. Beside this classical and well-studied pathway, a number of papers are dealing with the role of epigenetic mechanisms in the response to environmental xenobiotics. In this review, we report on the potential role of epigenetic mechanisms in dioxins-induced cellular response by inspecting recent literature and focusing our attention on epigenetic mechanisms induced by the most toxic 2,3,7,8-TCDD.

## 1. Introduction

Dioxins are highly toxic and persistent organic pollutants, distributing throughout the environment and accumulating in the food chain, mainly in the animal’s fatty tissues. They can cause cancer, reproductive and developmental issues, damage to the immune system, and can deeply interfere with the endocrine system [1,2,3,4]. Dioxins are principally released into the atmosphere as undesired products of various combustion and industrial processes [5], distributing into environmental matrices such as soil and water. From these matrices, dioxins accumulate in plant and animal tissue until they accumulate in human tissues through food chain biomagnifications processes. In this frame, it is important to emphasize that the half-life of TCDD in humans is very long; it has been estimated in the range between 7.1 [6] and 11.3 years [7].

Due to their high toxicity and environmental persistence, many efforts for real-time monitoring of these organic pollutants have been realized [8,9,10], unfortunately, the objective of constant monitoring of the potential sources of emission and the possibility of recognizing each single conformer in a complex mixture has not yet been fulfilled.

These halogenated organic compounds belong to two families i.e., Polychlorinated Dibenzo-*p*-Dioxins (PCDDs) and Polychlorinated Dibenzo Furans (PCDFs). All dioxins consist of two benzene rings connected by one or two oxygen atoms; they can bind between four to eight chlorines atoms, generating a wide family of conformers (75 PCDDs congeners and 135 PCDFs congeners) whose molecular reactivity toward cellular targets can change dramatically determining different levels of toxicity. Among these 210 congeners, the 2,3,7,8-tetrachlorinated (TCDD) species have the highest toxicity. Toxicity drastically decreases when adding non-lateral chlorines or removing lateral chlorine from the two aromatic rings [11]. This behavior can be explained in terms of ligand–receptor binding affinities [12,13] between the particular congener and AhR receptor, a signal transducer protein which is responsible of the various biological effects following the ligand–receptor interaction. There are 7 laterally chlorinated PCDDs and 10 laterally chlorinated PCDFs. The toxicity equivalent factor (TEF) [14] expresses the toxicity of dioxins and furans in terms of the most toxic form of dioxin, the 2,3,7,8-TCDD, whose TEF value is set to 1. 

The proposed molecular mechanism of PCDD/Fs envisages the binding of the dioxin to the cytosolic AhR which is a transcriptional regulator of cell growth, differentiation and migration. 

Dioxins are responsible for a wide variety of toxic and biochemical effects via AhR-mediated signaling pathways. The mechanism includes AhR binding of the toxic ligand in the cytoplasm and the following translocation of the receptor to the nucleus. It was also demonstrated that the heat shock protein HSP90 is capable of interacting with the AhR in cytoplasm. The HSP90/AhR interaction has the functional role of maintaining the cytosolic receptor conformation so that it is able to bind TCDD over the time. Upon ligand binding, the AhR undergoes a conformational change in the PAS A (Per-Arnt-Sim) domain, which facilitates its nuclear translocation and its consequent dimerization with the Ah receptor nuclear translocator (Arnt). The conformational change in the PAS A domain is concomitant with the dissociation from HSP90 protein and allows the conserved nuclear localization sequence in the N-terminal (42 amino acids) to stimulate the nuclear translocation of the AhR-TCDD complex, which is further facilitated by importins. After the translocation of AhR in the nucleus, there is its dimerization with Arnt and this new formed heterodimeric transcription factor binds to Xenobiotic-Responsive Elements (XRE) [15] upstream the promoter of *CYP1A1* gene (coding for cytochrome P-450 1A1) and other Ah-responsive genes that regulate the expression of the genes involved in polycyclic aromatic hydrocarbon metabolism and detoxification [16]. 

P-450 1A1 is one of the main xenobiotic metabolizing enzymes and TCDD is one of the most potent inducers of the *CYP1A1* gene. Following transcriptional activation, AhR is released from DNA and is rapidly exported from the nucleus and degraded via the ubiquitin-mediated 26S proteasome pathway [17]. 

Recently, several genome-wide transcriptional studies have identified numerous AhR target genes that lack the canonical XRE recognition site in the promoters region. Characterization of one of these target genes, i.e., the Plasminogen Activator Inhibitor 1 (*PAI1*), led researchers to discover a novel Nonconsensus XRE (NC-XRE) recognition site which confers to TCDD-AhR complex the possibility to stimulate gene expression independently from the presence of the Arnt [18]. Further experiments demonstrated that AhR binding to the NC-XRE depended on the interaction with Kruppel-Like Factor 6 (KLF6) [19]. Immunoprecipitations and in vitro DNA binding studies have demonstrated that the AhR and KLF6 proteins form the heterodimer necessary for the binding to the NC-XRE sequence. The heterodimer formation involves the interactions between the AhR C terminus and KLF6 N terminus, respectively. Wilson and coworkers reported that the capability of binding NC-XRE depends on the 5’ basic region in KLF6 [19]. These new results show a novel AhR signaling mechanism distinct from the canonical XRE-based process that will open new scenarios in the future understanding of AhR biology. Besides the pathways reported above it is known that TCDD-induced AhR may also be involved in cell cycle regulation, mitogen-activated protein kinase cascades (MAPK), immediate-early gene induction and in cross-talks with other nuclear receptors.

It has been reported that EGFR (Epidermal Growth Factor Receptor), activated by TCDD, plays a role in developmental toxicity and hepatocarcinogenecity induced by dioxin [20]. Also, the cytosolic signaling proto-oncogene tyrosine protein kinase c-Src can be activated by TCDD, as demonstrated in experimental animal models where its activation contributes to TCDD toxicity [21]. Moreover Tomkiewicz and coworkers have shown that the AhR, through the interaction with Src, activates focal adhesion kinase (FAK) promoting integrin clustering and cell migration [22]. Park et al. also reported that TCDD interacts with MAPK pathway-activating ERK and p38 in a mouse macrophage cell line [23]. Furthermore, TCDD is responsible for the promotion of the physical interaction between the AhR and pRb (Retinoblastoma protein). Two distinct models have been proposed, one supporting the AhR-pRb interaction acting in the co-repression of the transcription factor E2F activity and the other which entails AhR-pRb interaction and participation in transcriptional co-activation of genes encoding G_1_ phase-regulatory proteins [24]. Many studies have also reported the cross-talk between AhR and estrogen receptor (ER) signaling pathways. Arnt may be recruited by estrogens and increases ER-mediated gene transcription independently of AhR, but on the other hand, the AhR-Arnt complex may also activate ER-dependent genes in the absence of ER agonist [25,26].

AhR may also interact with the nuclear transcription factor NF-κB (Nuclear Factor kappa-light-chain-enhancer of activated B cells) by regulating inflammation in the lung and other tissues [27]. 

AhR activation also increases the stability of Nrf2 (Nuclear factor-erythroid 2-related factor 2), a transcription factor which induces the expression of antioxidants and phase II enzymes, i.e., detoxifying enzymes, thus amplifying the induction of genes encoding enzymes involved in the detoxification and the elimination of xenobiotics [28].

The biochemical and toxic effects of TCDD and related compounds substantially vary between different species, strains, target tissue or organ, age, and sex [29]. Exposure to 2,3,7,8-TCDD induces chloracne in humans and in certain strains of hairless mice but not in other animal species [16,30]. Long-term studies indicate that in some strains of rats an induction of hepatocellular carcinomas only in females has been observed. In humans, long-term exposure has been associated to immune suppression. The wide range and variability of both long and short-term effects suggests that dioxins act as Selective AhR Modulators (SAhRMs) [29,31]. 

AhR/Arnt complex can indeed interact with several cofactors and consequently AhR activity can be modified throughout the cell cycle reflecting the nature of the interaction with each specific recruited cofactor. 

Cofactors recruitment in both XRE and NC-XRE pathways has a direct impact on epigenetic changes associated with AhR-mediated gene expression. 

In this review, we will focus on the recent literature dealing with epigenetic mechanisms induced by 2,3,7,8-TCDD, considering three main epigenetic mechanisms, i.e., DNA methylation, histone modifications and non-coding RNAs (ncRNAs).

## 2. Brief Overview on Epigenetic Mechanisms

Environmental epigenetics is the study of the molecular mechanisms which takes place between environmental factors and the epigenome capable of altering the biology of organs, tissues and cells, resulting in adaptive evolutionary changes and/or diseases development [15].

Disease susceptibility depends on a complex interplay between the individual genetic profile and epigenetic modulations induced by endogenous or exogenous environmental factors [32,33]. Epigenetics is characterized by reversible, heritable modifications which are mitotically stable [34] and do not involve alterations in DNA sequence.

**Figure 1:**
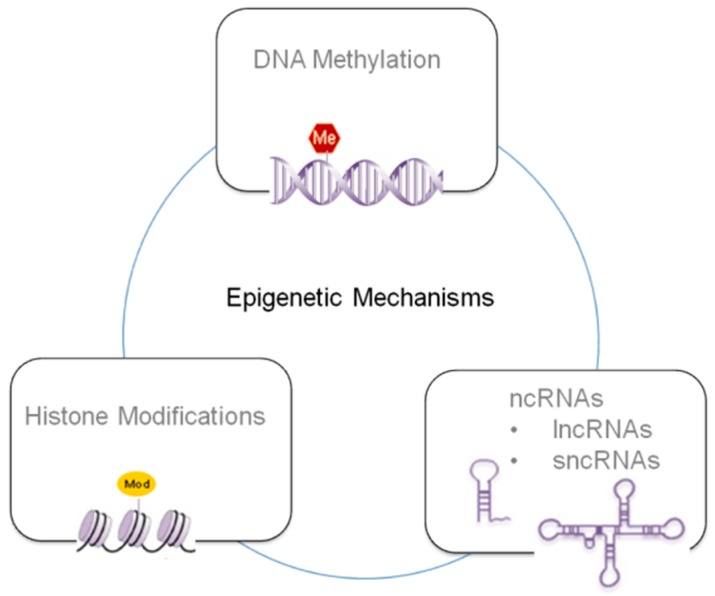
Schematics of the main epigenetic mechanisms involved in the xenobiotics response.

Epigenetic mechanisms can be summarized in three principal classes depending of the specific mechanism and target (see Figure 1), i.e., DNA methylation, histone modifications and ncRNAs [35,36,37]. These strongly interlaced processes determine if a gene can be expressed or silenced, playing a crucial role in gene expression and cellular development through complex mechanisms that can affect transcript stability, DNA folding, nucleosome positioning, chromatin compaction, and nuclear organization.

DNA methylation is a covalent modification of DNA by enzymatic addition of methyl groups to the 5-position of cytosine bases. For vertebrates, the CG dinucleotide sequence is one of the principal targets of DNA methylation because it is preferentially recognized by DNA methyltransferases (DNMTs) which use S-adenosylmethionine [29] as a methyl group donor. Because this sequence is a palindrome, the methylation pattern can be copied during cell division onto the daughter strand. In this way, DNA methylation becomes heritable and capable of generating long-term changes in gene expression in daughter cells and generations of animals [38,39,40]. DNA methylation of regulatory regions, such as promoters, acts by silencing gene expression principally by preventing the identification and access of transcription factors to their own consensus sequences [41]. 

In addition to 5-methylcytosine, mammalian DNA contains also low levels of various modified DNA bases, such as the 5-hydroxymethylcytosine, arising from DNA damage, which are generally eliminated by DNA repair processes. Anyway, Tahiliani et al. discovered the existence of the enzyme TET1 (ten-eleven translocation protein) 5-methylcytosine oxidase catalyzing the formation of 5-hydroxymethylcytosine from 5-methylcytosine [42].

It has been proposed that 5-hydroxymethylcytosine might have a role in direct DNA demethylation. The oxidation of 5-methylcytosine at methylated CpG sites is known to inhibit the binding of the methyl-CpG binding domain of the methyl-CpG binding protein 2 (MeCP2), which is a transcriptional repressor, suggesting a potential regulatory role of 5-hydroxymethylcytosine [43]. Moreover, the deamination of 5-hydroxymethylcytosine, producing 5-hydroxymethyluracil and generating a mismatched base pair between 5-hydroxymethyluracil and guanine, might promote DNA demethylation by the potential DNA repair mechanisms [44].

Another kind of epigenetic process is histone modifications at specific sites. 

In eukaryotic cells, genomic DNA is packaged into chromatin by four core histones (H2A, H2B, H3, and H4) and the linker histone (H1). The main chromatin modifications are histone acetylation and deacetylation at specific Lysine residues which dynamically activate or inhibit gene expression remodeling chromatin, thus making accessible or not specific genomic sites.

Acetylation usually stimulates gene activity and depends on chemical reactions catalyzed by enzymes i.e., histone acetyltransferases (HATs), histone deacetylases (HDACs) and by acetyl-coenzyme A (AcCoA) [15,45]. Deregulation of HAT/HDAC balance is associated with developmental defects, disease and cancer. On the contrary, histone methylation, promoted by histone methyltransferase (HMT), acts by silencing or activating specific genes depending on the methylation sites. AcCoA and SAM are derived from metabolism, for this reason, histone modification can be influenced by metabolism, diet and toxicants. 

Histone phosphorylation takes place principally but not exclusively on serines, threonines and tyrosines in the N-terminal of histone tails. The levels of these modifications are controlled by kinases and phosphatases that add and remove the phosphate groups respectively [45].

Another kind of histone modification is the deimination, a reaction which involves the conversion of arginine in citrulline.

Other histones modifications include ADP ribosilation on glutamate and arginine residues, ubyquitilation and sumoylation [45]. 

The ncRNAs derive from non-coding regions of the mammalian genome which are transcribed and processed into small and large non-coding RNA molecules [46]. ncRNAs with a size >200 nucleotides have been classified as large non-coding RNAs (lncRNAs), while ncRNAs <200 nucleotides represent the so called small non-coding RNAs (sncRNAs). These ncRNAs can operate as epigenetic regulators by interacting directly with epigenetic factors involved in chromatin remodeling (DNA methylation, histone modifications), thus promoting the regulation of complex gene networks [35]. Furthermore, lncRNAs can indirectly regulate epigenetic states by affecting transcriptional or translational activity or by affecting the stability of mRNAs encoding for epigenetic factors [47,48]. Some lncRNAs can also attract chromatin remodeling complexes to obtain local gene silencing through DNA methylation, or repressive histone modifications [35]. Altered expression profiles of lncRNAs have been observed after exposure to environmental chemicals. Exposure to these chemicals, such as benzopyrene, benzene, cadmium, and also TCDD has been correlated to alterations in lncRNAs expression both in vitro and in vivo. Deregulated lncRNAs can also alter the expression of target genes directly or indirectly via regulating the level of microRNAs (miRNAs) [49].

miRNAs are small (18–22 nucleotides), highly conserved, ncRNAs synthesized in the nucleus, maturating in the cytoplasm. They regulate gene expression by binding to complementary sequences on target mRNAs determining translational repression or target degradation with consequent gene silencing [50]. The analysis of miRNAs has highlighted their importance in the regulation of target genes expression and their involvement in both physiologic and pathologic scenarios. Several studies have demonstrated an association between deregulation of miRNAs and exposure to environmental chemicals, among them the dioxins [51].

### 2.1. TCDD and DNA Methylation

In the last years, many studies have shown an association between TCDD exposure and altered DNA methylation in mice. Singh et al. [52] have demonstrated that the administration of TCDD to C57BL/6 mice models, in which acute colitis was induced by dextran sodium sulphate (DSS) treatment, is able to block colitis symptoms through the induced CpG methylation at specific sites in the promoters of various genes present in murine T cells. In particular, a single dose of 25 μg·kg^−1^ of TCDD reduced the colitis symptoms through the activation of the AhR pathway, leading to a differential expression of the pathology-involved genes, i.e., *Foxp3* and *IL-17*. The increased methylation of CpG islands (CpGs) of *Foxp3* promoter and the demethylation of CpGs of *IL-17* promoter, due to acute colitis induced in C57BL/6 mice, were reversed after TCDD treatment.

Further in vitro experiments with activated T cells from C57BL/6 mice and methylation PCR assays confirmed that TCDD induces partial demethylation in *Foxp3* promoter and hypermethylation of the *IL-17* promoter, as assessed by band intensities of demethylated and methylated primers. 

Wu et al. [53] instead reported a study on DNA methylation changes due to dioxin exposure in Jcl:ICR mice embryos. The expression of the *H19* gene was significantly decreased, while the *IGF2* gene expression was reduced, but not significantly. Methylation analysis of the *H19/IGF2* imprint control region, using bisulfate genomic sequencing, determined that more CpGs from the TCDD-treated group were methylated than those from controls. Furthermore, the 5’ promoter region showed a higher proportion of methylated CpGs in TCDD-treated embryos compared to controls. DNMT activity increased in TCDD-exposed targets, indicating its possible role in altering the methylation pattern after TCDD exposure.

In a recent study, Wang and coworkers [54] investigated the effects of TCDD on the global and CpGs DNA methylation status and the expression of DNA methyltransferases levels in palate tissue of fetal mice. Pregnant C57BL/6J mice were administered with corn oil or TCDD 28 μg/kg at gestation day 10.5. After being exposed to TCDD, embryonic palate tissue showed a decrease in the methylation level of site 2 of CpGs in the promoter region of *DNMT3a*, correlated to the over-expression of DNMT3a. Furthermore, the authors noted a significant change in DNA methylation level on gestation day 13.5, resulting from the over-expression of DNMT1 and DNMT3a. These findings suggest that methylation and the over-expression of DNMT1 and DNMT3a could be the epigenetic mechanisms that cause palate malformation in fetal mice induced by maternal exposure to TCDD. 

A couple of recent papers have reported on the toxic developmental effects of TCDD in embryos, larvae and adult zebrafish [55,56]. Both the studies mentioned highlighted that TCDD did not affect the overall amount of 5-methylcytosine during development, but specific methylation of the CpGs in the promoter of AhR target genes was either unchanged or differentially affected. In particular, hypomethylation was observed in 11 out of 22 CG dinucleotides within the *cfos* promoter, while 14 out of the 34 CG sites were hypermethylated in the *ahrra* promoter [56]. These alterations have been correlated to TCDD-induced deregulation of *dnmt* gene expression [56]. The authors reported that TCDD exposure during early embryogenesis determined developmental stage-specific up-regulation of *dnmt1* and *dnmt3b2* and the down-regulation of *dnmt3a1*, *dnmt3b1* and *dnmt3b4* [56]. These findings strongly suggest that TCDD could impact both the establishment and maintenance of DNA methylation patterns of genomic loci not necessarily restricted to AhR targets.

Amenya and coworkers in a recent paper reported that treatment with TCDD induced the demethylation of two CpGs at the *Cyp1a1* proximal promoter (−500 and −420) within 24 h in C57BL/6J mice liver [57]. The authors noted that *Cyp1a1* transcriptional activation preceded *Cyp1a1* promoter demethylation, indeed *Cyp1a1* mRNA was detectable in 6 h, which is earlier than CpG demethylation which was observed 24 h later. The authors observed a gradual decline in 5-methylcytosine and 5-hydroxymethylcytosine at the same CpGs (−420), indicating a progressive demethylation that involved the transition of 5-methylcytosine to 5-hydroxymethylcytosine intermediate typical of the Tet-mediated demethylation process. In support of Tet proteins involvement in Ahr-dependent active demethylation, the authors observed the Ahr-dependent occurrence of Tet3 at the *Cyp1a1* promoter. Additionally, Tet2 and Tet3 knockdown suppressed dioxin-induced demethylation in an artificially-methylated promoter. ChIP assay of the base excision repair proteins (BER) revealed that Apex1 and thymine DNA glycosylase (Tdg) also occurred at the *Cyp1a1* promoter in an Ahr-directed manner, although only Tdg knockdown inhibited dioxin-induced demethylation of the methylated plasmid [57].

The previously mentioned studies, summarized in Table 1, have determined that dioxin exposure is responsible for altered DNA methylation in the cell lines and animals tested. It must be taken into account that all the studies here reported are limited to one or two generations, and therefore the heritability of the epigenetic changes remains to be determined with further studies.

### 2.2. TCDD and Histone Modifications

In 2010, Beedanagari et al. [36] studied the epigenetic role of TCDD in regulating the expression of *CYP1A1* and *CYP1B1* genes using human breast cancer cell line MCF-7 and Human hepatic cancer cell line HepG2 cells. Chromatin immunoprecipitation assays revealed that 100 nM TCDD increased histone modifications in the promoter regions of *CYP1A1* and *CYP1B1* in MCF-7 and in HepG2 cells. Histone acetylation was found at the levels of Lys 9 and 14 of histone H3. Trimethylation of H3 and acetylation of H4 at the level of Lys 4 were also found. The extent of each histone modification at the *CYP1A1* promoter was greater in HepG2 cells than in MCF-7 cells. On the contrary, the extent of the modifications at the *CYP1B1* promoter was much less in HepG2 cells then in MCF-7 cells both before and after dioxin treatment [36]. The above-mentioned histone modifications are generally associated with the activation of gene transcription. In this regard, CYP1A1 mRNA is highly induced by dioxin in both the MCF-7 and HepG2 cell lines, but CYP1B1 mRNA, which is strongly induced in MCF-7, is not induced in HepG2 cells. The authors demonstrated that in MCF-7 cells, after dioxin treatment, histone modifications increased at the *CYP1B1* promoter as a consequence of the recruitment of transcriptional coactivator p300 responsible for catalyzing direct histone acetylations and thus for contributing to maximal dioxin induction of *CYP1B1*, as also previously observed [58]. On the other hand, in HepG2 cells, dioxin failed to induce the recruitment of RNA Polymerase II or the TATA binding protein (TBP), acetylations of histones 3 and 4, or methylation of histone 3 at the promoter, despite the coactivators recruitment at the *CYP1B1* enhancer. The authors attributed this different behavior to the higher grade of CpGs methylation at the promoter of *CYP1B1* in HepG2 cells with respect to MCF-7 cells, which prevent the recruitment of TATA-binding protein (TBP) and RNA Polymerase II [36].

In a precedent study, Okino et al. demonstrated that TCDD led to the increasing of histone acetylation at H3 and H4 within *CYP1A1* promoter in human prostate cell lines PWR-1E and RWPE-1 [59]. The authors ascribed this chromatin modification to the heteromeric transcription factor AhR-Arnt, which recruits proteins having histone acetyltransferase activity. In the same study, the authors also detected an increased histone acetylation in the heavily methylated DNA region upstream of the regulatory elements of *CYP1A1* due to TCDD exposure [59].

Recently, Yuan et al. studied the effects of DNA methylation and histone acetylation on TCDD-induced cleft palate in fetal mice (C57BL/6J mice) [60]. Mice were treated with 28 µg/kg of TCDD on gestation day 10 while the control group was treated with an equal volume of corn oil.

Their results indicate that TCDD-induced cleft palate at the critical period of palate fusion (gestation days 13.5–14.5) is characterized by increased Transforming Growth Factor β (*TGF-β3*) gene expression, increased HAT activity and H3 iper-acetylation [60]. However, the underlying mechanisms for the deregulation of HAT remain to be fully understood and determined. 

2,3,7,8-tetrachlorodibenzo-p-dioxin is a carginogen involved in hepatocellular carcinoma (HCC) development. Due to its high-affinity for AhR, TCDD is able to promote a strong AhRC transcriptional activation.

Very recently, Wang et al. [61] studied the relationship between AhR activation and the expression of HDAC8 in patients with HCC. The authors stated that long-term exposure to environmental pollutants and its metabolites can activate AhR in the liver, causing the expression of HDAC8, resulting in an imbalance between acetylation/deacetylation with consequent tumorigenesis. To test if the inhibition of the tumor suppressor gene *RB1* and to test if HDAC8 expression was directly affected by *AhR*, the authors used hepatocytes isolated from *AhR*-wild type and *AhR*-null mice that were treated with TCDD. The authors found increased HDAC8 and decreased Rb1 expression in dioxin-treated *AhR*-wild type primary hepatocytes but not in those from *AhR*-null mice, demonstrating the role of AhR in determining HDAC8 over-expression and, consequently, the down-regulation of the tumor suppressor gene *RB1*. The modulation regulatory effect of *RB1* by HDAC 8 was also determined by using hepatoma cells with ectopic *HDAC 8* expression but transfected with *AHR* shRNAi. The hepatoma cells (SK-Hep1) with ectopic HDAC 8 expression reversed the enhancing effects of AHR knockdown on both the expression of RB1 and p53. These very interesting results indicate that *HDAC8* is a direct physiological target of *AhR* and that Rb1 in turn regulates cellular proliferation as a consequence of AhR activation.

The evidence of histones deacetylation on tumor-suppressive genes suggests that HDAC is involved in tumor formation and progression. Treating hepatocellular carcinoma with HDAC inhibitors can, at least partially, repress tumor proliferation and transformation by promoting the expression of tumor-suppressive genes [62]. It is worth noting that also mutations in histone-modifier enzymes are recurrently found in many disease states including cancer.

In a recent study, Joshi et al. demonstrated that AhR binding to the NC-XRE in response to 2,3,7,8-tetrachlorodibenzo-*p*-dioxin resulted in a concomitant recruitment of Carbamoyl Phosphate Synthase 1 (CPS1) to the NC-XRE site in cultured C57BL6 mouse primary hepatocytes [63].

CPS1 is a nuclear protein and in the hepatocytes is responsible for carbamylation (homocitrullination) of a functionally important histone H1 lysine residue (H1K34) implicated in transcriptional regulation. The authors thus demonstrated that homocitrullination is a novel epigenetic mark promoting chromatin remodeling into a more accessible, transcriptionally-active conformation. CPS1 promotes enhanced expression of the Peptidylarginine Deiminase 2 gene (*PADI2*), which encodes for the chromatin-modifying protein Peptidylarginine Deiminase 2 (PADI2) [63]. PADI2 has been shown to citrullinate both histone H3 and H4 [64]. Table 2 shows a summary of the TCDD-induced histone modifications above described.

### 2.3. TCDD and ncRNAs

Few studies concerning the role on ncRNAs in determing epigenic toxicity of TCDD are present in literature. Non-coding RNAs do not contain functional open reading frames, cannot encode proteins, and are transcribed from the entire genome [65]. Several lncRNAs have a critical role in embryonic development, while the dysfunction of some lncRNAs has been associated with birth defects and cleft palate. Moreover, these transcripts may play an important functional role in cancer biology [65].

Recently, Gao et al. [66] demonstrated the possible role of lncRNA H19 in the pathogenesis of cleft palate induced by TCDD in a mice model. The authors found that the expression levels of lncRNA H19 and its target gene *IGF2* presented specific embryo age-associated differences during the entire development of the pathology. In particular, the expression levels of lncRNA H19 varied with the stages of TCDD-induced palatogenesis between embrionyc days 13.5 and 15.5, that are important for the development of palate. The authors found that the expression levels of lncRNA H19 in TCDD-treated mice were lower on embryonic days 13.5 and 15.5 compared with those of the control, while a high expression was observed on day 14.5. Therefore, lncRNA H19 is suggested to be the primary contributor to the development of cleft palate induced by TCDD.

Moreover, the same authors observed an inverse correlation between lncRNA H19 and *IGF2* expression levels, i.e., the expression levels of *IGF2* gene were found to be higher on embryonic days 13.5 and 15.5, and lower on 14.5, thus showing an opposite expression trend with respect to lncRNA H19. These findings suggest that lncRNA H19 may function through the interaction with *IGF2* [66]. Anyway, the precise mechanisms through which lncRNA H19 regulates TCDD-induced cleft palate requires further investigation to elucidate the exact molecular mechanisms.

The number of papers on the roles of ncRNAs is fortunately growing and they will provide new insights into the toxicology induced by environmental chemicals. Different kinds of these environmental pollutants, including organic chemicals [67], heavy metals [68] and so on, have been demonstrated to disturb the expression of ncRNAs. Consequently, the altered levels of ncRNAs lead to a change of the target miRNAs and mRNAs, thus interfering with the normal signaling pathways, which in turn can induce pathological phenotypes. 

Furthermore, TCDD-mediated alterations in miRNA expression may be involved in cancer, hepatic injury, apoptosis, and cellular development [69]. 

From the literature, it is not clear how the toxic effects of TCDD are regulated by certain miRNAs. In particular, the possibility that miRNAs might modulate mRNA levels and consequently TCDD-induced toxicity has not been fully explored and understood. An important study by Tsuchiya and coworkers reported that the lower expression of miRNA-27b due to AhR-regulated genes increased CYP1B1 levels [70]. CYP1B1 protein is abundant in cancerous tissues. The authors identified a matching sequence with miR-27b in the 3′-untranslated region of human CYP1B1. Luciferase assays revealed that the reporter activity of the plasmid containing the miR-27b recognition element was decreased in MCF-7 cells but not in Jurkat cells. The authors found that exogenously-expressed miR-27b decreased the luciferase activity in Jurkat cells, while in MCF-7 cells the antisense oligoribonucleotide for miR-27b was able to restore the luciferase activity, increasing the protein level and the enzymatic activity of endogenous CYP1B1. These results led the authors to the conclusion that human CYP1B1 is post-transcriptionally regulated by miR-27b. The authors also analyzed the expression levels of miR-27b and CYP1B1 protein in breast cancerous and adjacent noncancerous tissues from 24 patients. They found in most patients that the expression level of miR-27b was decreased in cancerous tissues, accompanied by a high level of CYP1B1 protein. The decreased expression of miRNA-27b has been suggested to be one of the causes of high expression of CYP1B1 protein in cancerous tissues [70]. This is the first study which highlights the role of miRNAs in regulating not only essential genes, but also drug-metabolizing enzymes.

In another study, it has been reported that treatment with TCDD in vivo caused few changes in miRNAs levels in mouse or rat livers; those changes that were statistically significant were of modest magnitude [71]. The authors of this study used miRNA array platforms and quantitative reverse transcriptase–PCR to measure miRNA levels in wild type versus *Ahr*-null mice, in dioxin-sensitive Long-Evans (L-E; Turku/AB) rats *versus* dioxin-resistant Han/Wistar (H/W; Kuopio) rats and in rat 5L and mouse Hepa-1 hepatoma cells in culture. 

Treatment with 2,3,7,8-tetrachlorodibenzo-p-dioxin in vivo caused few changes in miRNA levels in mouse or rat livers, and those changes that were statistically significant were of modest magnitude. Hepatoma cells in culture also exhibited few changes in miRNA expression levels in response to TCDD. Only few miRNAs, and especially miRNA 122a, differed in expression between *Ahr*-null mice compared to mice with WT AHR or between L-E rats (that have WT AHR) compared to H/W rats (whose AHR has a large deletion in the transactivation domain). The authors concluded that it is unlikely that mRNA down-regulation is mediated by dioxin-induced miRNAs and that miRNAs do not play a significant role in dioxin toxicity in adult rodent livers [71]. Thus, it is likely that the AhR-agonist-mediated changes in miRNAs may be organ-specific.

Singh and coworkers [69] demonstrated instead that exposure to TCDD during pregnancy can have a significant effect on the miRNAs profile of fetal thymus and can influence the regulation of a large number of genes that may affect the development of the immune system. The authors found down-regulated expression of the miRNAs 27a, 28, 29, 182, 203 and 290 in fetal thymi following TCDD exposure. The authors stated that these miRNAs may regulate the expression of TCDD-modulated genes; indeed these miRNAs possessed a highly complementary sequence with a 3′UTR region in the *AhR* gene. The downregulated expression of these miRNAs suggests that they may be involved in further pathways inducing the AhR in fetal thymi. Similarly, the authors also observed the downregulated expression of some other miRNAs (31, 101b, and 335) in fetal thymi post-TCDD exposure. Using the microRNA.org database for the prediction of miRNAs targets, they observed that these miRNAs possessed a highly complementary sequence for the 3′UTR of *CYP1A1* gene, which may explain the ability of TCDD to induce *CYP1A1* gene through this epigenetic mechanism [69]. All the ncRNAs effects, induced by TCDD exposure on target genes, discussed in this paragraph are shown in Table 3.

New studies on the TCDD-induced epigenetic modulation through ncRNAs in vivo and in vitro will have fundamental importance in order to better understand TCDD-mediated toxicity and to find alternative molecular pathways.

## 3. Conclusions and Future Perspectives

A growing body of literature is demonstrating the sensitivity of the epigenome to environmental pollutants such as TCDD in a variety of cells, tissues and animal models. Although molecular pathways involved in the alteration of an epigenetic landscape in response to dioxins are largely unknown, several mechanisms of AhR-dependent histone modification, DNA methylation and ncRNAs regulation mechanisms have been described. 

Epigenetic mechanisms represent an important way through which the genome responds to the environment, leading progressively to permanent changes in gene expression, affecting the phenotypes. It is important to point out that TCDD is usually found in the environment as a component of complex pollutants mixtures, and this situation is far from the laboratory exposure conditions and the evidence from human-based epidemiological observation cannot often be directly related to the results obtained from the animal models. Nevertheless, the development of new sequencing and biochemical techniques are allowing researchers to access the epigenome to understand the interfaces between epigenetics, TCDD exposure and disease-related phenotypes. These new scientific approaches will allow for a systematic analysis of the signaling pathways and molecular mechanisms and how they are linked to environmental exposure. In this frame, further studies are required, not only for the understanding of how environmental signals are translated into epigenetic changes, but also to understand if they are transmitted to the germ-line and thus to the offspring. In particular, it is important to figure out if epigenetic modifications reprogramming is transient, persists or if it is further modified throughout the course of the life of the organism, and how environmental factors are able through germ cell epigenetic modification to be permanently transmitted across the generations. It is worth noting that the three epigenetic mechanisms above described are intertwined and thus are able also to jointly affect transcript stability, DNA folding, nucleosome positioning, chromatin compaction, and ultimately nuclear organization in the framework of very complex interaction networks.

For instance, histone deacetylation and methylation at specific amino acid residues contribute to the establishment of DNA methylation patterns. miRNAs expression is controlled by DNA methylation in miRNAs encoding genes, and, in turn, miRNAs have been shown to modify DNA methylation. Future studies that include comprehensive investigations of multiple epigenetic mechanisms might help understand the timing and participation of DNA methylation, histone modifications and miRNAs in determining the environmental effects on disease development.

In the last years, a wide range of new molecular biology techniques have allowed for the comprehension of the complex scenario of the epigenetic molecular basis. Among these techniques there are chromatin immunoprecipitation, fluorescent in situ hybridization, methylation-sensitive restriction enzymes, DNA adenine methyltransferase identification (DamID), and bisulfite sequencing. Furthermore, computational epigenetics, taking advantage of bioinformatics methods, will fulfill an important role in unraveling these interlaced molecular networks. 

An increasing number of clinical and preclinical studies have shown that most of the epigenetic changes are reversible. These findings offer novel insights to develop new preventive and therapeutic strategies taking advantage of molecules that modify the activities of epigenetic-involved enzymes and molecules such as DNMTs and HDACs and of miRNA. Several drugs able to stimulate histone acetylation and DNA hypomethylation have been designed and developed in order to restore the normal transcription level of genes. In this view, future epidemiology studies will have the fundamental role of evaluating the effects of environmental exposure on the epigenome, how these effects are influenced by the interaction with other risk factors, and how they can be mitigated by the correct lifestyles or by new drugs.

From a general point of view, future researches should also be addressed to environmental xenobiotics efficient monitoring in order to reduce exposure and to prevent chronic and acute diseases. 

## Figures and Tables

**Table 1:** Summary of the recent papers dealing with new insights in TCDD-induced epigenetic Methylation/Demethylation of target genes.

Model	Target Genes	Epigenetic Mechanism: DNA Methylation/Demethylation	Refs.
Activated T cells from C57BL/6 mice	*Foxp3* and *IL-17*	Dymethylation of CpGs of *Foxp3* promoter; Hypermethylation of *IL-17* promoter.	[52]
Jcl:ICR mice embryos	*H19* and *IGF2*	Hypermethylation of CpGs of *H19* and *IGF2* promoters; Over-expression of DNMT.	[53]
Palate tissue of fetal C57BL/6J mice	*DNMT3a*	Dymethylation of CpGs in *DNMT3a* promoter; Over-expression of DNMT3a.	[54]
Zebrafish embryos	*cfos* and *ahrra*	Hypermethylation of CG dinucleotides of *cfos* and *ahrra* promoters; Up-regulation of *dnmt1* and *dnmt3b2*; Down-regulation of *dnmt3a1*, *dnmt3b1*, *dnmt3b2.*	[56]
Adult C57BL/6 mice Liver	*Cyp1a1*	Demethylation of CpGs of *Cyp1a1* promoter; *Cyp1a1* transcriptional activation.	[57]

**Table 2:** Summary of the recent papers dealing with new insights in TCDD-induced epigenetic histone modifications of target genes.

Model	Target Genes	Epigenetic Mechanism: Histone Modification	Refs.
Human breast cancer MCF-7 and human hepatic cancer HepG2 cell lines	*CYP1A1* and *CYP1B1*	Promoters of *CYP1A1* and *CYP1B1* of MCF-7 and HepG2 cell lines: Acetylation of Histone H3 (Lys 9 and Lys 14); Trimethylation of Histone H3; Acetylation of Histone H4 (Lys 4).	[36]
Human prostate cell line RWPE-1	*CYP1A1*	Acetylation of histone H3 and H4 in *CYP1A1* promoter; Histone acetylation upstream the regulatory elements of *CYP1A1* gene.	[59]
Fetal mice C57BL/6J	*TGF-β3*	Increased *TGF-β3* gene expression; Hyperacetylation of Histone H3; Up-regulation of HAT activity.	[60]
Hepatocytes isolated from *AhR*-wild type and *AhR*-null mice	*RB1*	Over-expression of HDAC8; Decreased expression of Rb1 tumor suppressor.	[61]
Cultured C57BL6 mouse primary hepatocytes	*PADI2* and *CPS1*	Homocitrullination by CPS1 of Lys 34 of histone H1; Enhanced expression of PADI protein with consequent histone H3 citrullination.	[63]

**Table 3:** Summary of the recent papers dealing with new insights in the role of ncRNAs in mediating TCDD toxicity.

Model	Target Genes	Epigenetic Mechanism: Non-Coding RNAs	Refs.
Kunming mice embryos	*IGF2*	Lower expression levels of lncRNA H19 in TCDD-treated mice between gestation days 13.5 and 15.5, associated with augmented expression of *IGF2* (on days 13.5 and 15.5); Higher expression levels of lncRNA H19 on gestation day 14.5 associated with a strong reduction of *IGF2* expression.	[66]
MCF-7 and Jurkat cells	*CYP1B1*	The expression of miRNA-27b strongly regulates the expression of CYP1B1 protein in cancerous cells and tissues.	[70]
WT, L-E, H/W, *AhR*-null mices and mouse Hepa-1 hepatoma cells	*CYP17a1*, *CYP7a1*, *Thrsp*, *Scd1*, *Tgfbp1i4*	Very little effects in lowering levels of few miRNA (101a, 138, 203, 361, 498, 542-5p), but especially miRNA 122a.	[71]
Fetuses Thymic cells (C57BL/6 mice)	*CYP1A1*	Down-regulation of miRNAs 27a, 28, 29, 182, 203, 290, 31, 101b, and 335.	[69]
